# What patients think about ICU follow-up services: a qualitative study

**DOI:** 10.1186/cc7769

**Published:** 2009-04-01

**Authors:** Suman Prinjha, Kate Field, Kathy Rowan

**Affiliations:** 1 DIPEx Research Group, Department of Primary Health Care, University of Oxford, Rosemary Rue Building, Old Road Campus, Headington, Oxford OX3 7LF, UK; 2 Intensive Care National Audit & Research Centre (ICNARC), Entrance A, Tavistock House, Tavistock Square, London, WC1H 9HR, UK

## Abstract

**Introduction:**

UK policy recommendations advocate the use of intensive care unit (ICU) follow-up services to help detect and treat patients' physical and emotional problems after hospital discharge and as a means of service evaluation. This study explores patients' perceptions and experiences of these services.

**Methods:**

Thirty-four former ICU patients were recruited throughout the UK, using maximum variation sampling to achieve as broad a range of experiences of the ICU as possible. Participants were interviewed at home by a qualitative researcher unconnected to their hospital care. Interviews were recorded and transcribed for analysis. We report a qualitative thematic analysis of patients' experiences of ICU follow up.

**Results:**

Former patients said they valued ICU follow-up services, which had made an important contribution to their physical, emotional and psychological recovery in terms of continuity of care, receiving information, gaining expert reassurance and giving feedback to ICU staff. Continuity of care included having tests and being monitored, referrals to other specialists and ICU follow-up appointments soon after hospital discharge. Information about physical, emotional and psychological recovery was particularly important to patients, as was information that helped them make sense of their ICU experience. Those without access to ICU follow-up care often felt abandoned or disappointed because they had no opportunity to be monitored, referred or get more information.

**Conclusions:**

Former patients value having ICU follow-up services but many found that their healthcare needs were unmet because hospitals were unable to provide the aftercare they required. Most participants were aware of the financial constraints on the health system. Although they valued ICU follow-up care, they did not want it to continue indefinitely, with many of them declining appointment invitations when they themselves felt they no longer needed them.

## Introduction

Most research on the recovery after hospital discharge of patients who were in the intensive care unit (ICU) has focused on their quality of life. Quantitative studies have concentrated on measuring the prevalence of different physical and psychological problems [1] and qualitative studies have focused on patients' physical and emotional experiences once they are back in the community [2,3]. We now have a valuable insight into the diversity of physical and psychological problems that patients can experience during recovery. These can last for months in some cases and several years in others [4], with some patients never returning to their previous level of health because ICU treatment can result in a reduced quality of life [5,6]. Common physical problems after discharge can include muscle weakness [7], breathlessness [8] and sexual dysfunction [9]. Psychosocial problems can include anxiety, depression [10], hallucinations, delusional memories and nightmares [11]. For many former ICU patients, returning home and convalescing can be the most psychologically stressful phase of critical illness [12].

The recovery trajectory for ICU patients is often prolonged and suboptimal, so follow up of patients surviving ICU treatment has been advocated [13]. In 1999 the UK Audit Commission recommended the provision of aftercare following an ICU stay [14] and, in 2000, the Department of Health recommended that all National Health Service (NHS) Trusts "review the provision of follow-up services and ensure there is appropriate provision for those who will benefit" [15], a stance echoed in its report *Critical Care Outreach *in 2003 [16]. The Department of Health also advised collecting follow-up data to evaluate these services.

ICU follow-up services have been advocated because they can help detect and therefore treat physical problems after hospital discharge, including weakness, weight loss, skin irritation and joint pains [17,18], and psychosocial problems, such as anxiety, depression and post-traumatic stress disorder (PTSD) [19]. Without these services, the only indication of outcome after ICU would be re-admission or visits by former patients and their relatives to the ICU, which are unreliable [20]. ICU follow-up services also allow data on mortality and health status after discharge to be measured which, with feedback from patients about health status and care, can be used to monitor and evaluate services [21]. However, despite recommendations, the clinical benefits and cost-effectiveness of ICU follow-up services are unclear. Current research aims to evaluate ICU follow-up services quantitatively in terms of patient benefit and cost-effectiveness [22].

The provision of ICU follow-up services in the UK remains inconsistent. The exact number of active ICU follow-up clinics is unknown, but a UK survey in 2006 estimated that 80 (30%) of the 266 ICUs that took part ran a follow-up clinic (response rate 89%) and those ICUs without one (158 ICUs, 88%) mostly cited 'financial constraints' as the reason [23].

In the UK, as elsewhere, ICU follow-up care is relatively new and still evolving. Studies conducted in the UK and Australia have reported on the establishment and development of ICU follow-up services, including challenges and benefits [24,25]. This work, some of it qualitative, has been conducted by the nurses responsible for setting up and running the service and a few have included 'elementary service evaluations' [26]. Cutler and colleagues recommend that future research should explore experiences of ICU follow up because little is known about patients' and relatives' perceptions and experiences of these services [27]. Maddox and colleagues stressed the futility of designing interventions without reference to patients' and carers' perceptions and preferences [3].

Although it is unclear whether ICU follow-up services change outcome, there is also a dearth of research into patients' perceptions and experiences of these services. This paper focuses on the experiences of ICU follow-up care from the perspective of former patients. It is unique in the field because it includes patients from many different ICUs across the UK and the research was conducted by an experienced qualitative researcher who was entirely unconnected to their care. This study is part of a larger project on patients' and relatives' experiences of ICU [28] and is not intended as a formal evaluation of ICU follow-up services. ICU follow-up care was an important theme in the overall illness narratives of patients as was, for example, their experience of transfer from the ICU to a general ward [29].

## Materials and methods

### The Healthtalkonline project (formerly DIPEx)

The Healthtalkonline website [30] is a resource based on narrative interviews about people's experiences of health and illness. All Healthtalkonline projects are conducted with multicentre research ethics committee approval. Each study consists of about 40 to 50 narrative interviews conducted one-to-one in the participant's home by an experienced qualitative researcher. All the interviews are digitally recorded (video or audio) and professionally transcribed. Healthtalkonline publishes its analysis and findings on its website, which is written primarily for a lay audience, and is also being used in healthcare and inter-professional medical education. The wider aims of Healthtalkonline are described in more detail elsewhere and also on the website [31-33]. The project on which this study is based was funded by Intensive Care National Audit and Research Centre (ICNARC) and conducted by SP, an anthropologist and member of the DIPEx research team, in 2005–06.

### The sample

Forty former ICU patients were interviewed in 2005 about their experiences of intensive care. Participants were recruited using a maximum variation sample in order to gain a broad range of experience of intensive care [34]. Maximum variation samples are used in qualitative interview studies to ensure a wide range of participants and experiences, not to be numerically representative. This means that the study may be generalisable in terms of the themes and issues that it identifies but to give frequencies would be misleading. Our sample included men and women from across the UK of different age groups and social and ethnic backgrounds. It also included participants who were admitted to the ICU as emergency and elective admissions, for different lengths of stay. An expert advisory panel of patient representatives, researchers and ICU clinicians helped us with sampling [35], advising us, for example, to include patients admitted for many different critical illnesses.

Reasons for emergency admission included pneumonia, pancreatitis, head injury, accidents, bowel perforation, aneurysm and surgical complications. Reasons for elective admission included surgery for various cancers and heart conditions. We continued interviewing patients until we were no longer adding new experiences to the analytic categories [36,37]. Of the 40 participants, five were in the ICU after elective surgery and one was a carer. Thirty-four were emergency ICU patients and it is their experiences that are the focus of this paper because, unlike elective patients, these patients have no prior warning of illness. They usually spend longer in both the ICU and ward and are therefore often weaker when they leave hospital (Table 1). All 34 participants had been treated in an ICU between 1994 and 2005 and were interviewed in their own home by SP. Participants discussed many aspects of their illness experience, from ICU admission to recovery, but here we focus specifically on their perceptions and experiences of ICU follow-up care (Table 2).

**Table 1:** Patients' biographies and experiences of formal care

**Interview** **number**	**Age** **(years)**	**Sex**	ICU/HDU stay *	Ward stay*	ICU follow-up care*
IC01	41	F	ICU: 11 days	2 weeks	Regular appointments with clinical psychologist
IC02	60	M	ICU: about 1 month	4 days	Attended initial appointments, declined future appointments
IC03	66	M	ICU: 5 weeks	5 weeks	1 appointment
IC04	46	F	ICU: about 5 weeks HDU: 1 week	Discharged after 1 week in HDU	2 appointments
IC05	40	F	ICU: 22 days HDU: 10 days	Just over a week	1 appointment
IC06	35	F	ICU: 3 weeks HDU: 36 hours	5 weeks	2 appointments and private counselling
IC07	60	M	ICU: 7 months	Ward: 1 month Home: awaiting rehab	None at time of interview
IC08	50	M	ICU: 10 days	Readmitted in 2006 and died	N/A
IC09	60	M	ICU: 3 days	10 days	No ICU follow-up
IC10	76	F	ICU: about 1 week	1 week	1 appointment
IC12	23	F	ICU: 21 days HDU: 2 weeks	2 weeks	Had been invited to attend 1^st ^appointment
IC14	35	F	ICU: 2 days	2 days, discharged herself	Declined appointment
IC15	38	F	ICU: 1 month, admitted 3 times in 2004	Several months on and off	No ICU follow-up
IC16	67	M	ICU: 8 weeks	3 weeks	No ICU follow-up
IC17	30	M	ICU: 12 days	2 weeks	No ICU follow-up
IC18	62	M	ICU: 18 days	5 weeks	2 appointments
IC21	72	M	ICU: about 4 weeks	1 week	No ICU follow-up
IC22	71	M	ICU: 17 days	A few days	At least 1 appointment
IC23	54	M	ICU: 17 days	2 weeks	1 appointment
IC24	44	F	ICU: 5 weeks	2 weeks	Physiotherapy referral after 1^st ^appointment, GP referral for counselling
IC25	45	M	ICU: 4 weeks	2 weeks	2/3 appointments
IC26	47	M	ICU: 30 days	8 days	No ICU follow-up
IC27	68	M	ICU: 2 weeks HDU: 2 weeks	Ward: 6 weeks; Rehab: 2 weeks	1 appointment
IC28	46	M	ICU: 7 weeks	4 weeks	1 appointment
IC29	47	F	ICU: 2 weeks HDU: 1 day	1 week	1 appointment, another expected
IC30	55	F	ICU: 6 days HDU: 5 days	Discharged after HDU	2 appointments
IC31	71	M	ICU: 2 weeks	3 weeks	At least one appointment
IC32	57	F	ICU: 29 days	Ward: 6 weeks Rehab: 6 weeks	3 appointments
IC34	37	M	ICU: 30 days total, admitted twice	Several months	At least one appointment
IC35	33	M	ICU: 17 days	Ward: 1 week Rehab: 3 months	At least one appointment
IC36	67	F	Could not remember		Awaiting 1^st ^appointment
IC37	58	M	ICU: 9 days	16 days	At least 1 appointment
IC38	55	F	ICU: 1 week	2 weeks	1 appointment
IC39	56	M	ICU: 4 days	3 days	No ICU follow-up

*Participants did not always know if or when they had moved from the intensive care unit (ICU) to step-down or high-dependency unit (HDU) care. Some did not know or could not remember how long they had spent on a ward. Participants were also not always sure how many ICU follow-up appointments they had attended.

F = female; M = male; N/A = not available; Rehab = rehabilitation.

The missing interview numbers are those of patients that were elective admissions.

**Table 2:** Patients' experiences of intensive care: main themes of the study

**Reasons for admission**
Emergency admissions
Planned admissions
Coming round and regaining consciousness
Sleep, dreams and hallucinations
Intensive care treatments
Physiotherapy
Emotional experiences in the intensive care unit (ICU)
Nursing care in ICU
Death and bereavement
High dependency units (HDU)

**Experiences in the general ward**
The general ward: care and environment
Physical and emotional experiences
Physiotherapy on the ward
Discharge and moving on

**Recovering at home**
Physical recovery
Emotional aspects of recovery
Making sense of what happened
Information
Information for people admitted to ICU for emergency treatment
Information: planned admissions
ICU follow-up care
Attitudes to life during and after recovery
Effects on family
Effects on work
Sources of support
Messages to others

### The interviews

We invited adult men and women throughout the UK to be interviewed about their experiences of intensive care for the Healthtalkonline website. Participants were recruited through health professionals, charities and support groups. People who wanted to take part contacted one of the researchers (SP), who discussed the project and demonstrated the website before the interview. We used in-depth narrative interviews to elicit people's stories and perspectives of being in the ICU. After the narrative, we used semi-structured questions so participants could elaborate on topics they had talked about earlier. The researcher could also, at this time, ask about issues that had arisen in previous interviews or been discussed in the intensive care literature to ascertain the importance of these topics to each participant. Interviews were conducted in people's homes and lasted up to two hours. People were encouraged to speak freely about their experiences for the benefit of other patients and relatives (via the Healthtalkonline website) and of health professionals in training. Healthtalkonline's research methods are described on its website [30].

Each interview was recorded on audio and video tape with the participant's consent and professionally transcribed. Participants were sent a copy of their transcript and biographical information to review. Once final copies of the transcripts and biographies had been agreed with the participants, they were asked to assign copyright to Healthtalkonline, thereby permitting us to use their interview extracts and biographical information on the Healthtalkonline website, as well as for research (including publications and teaching) and broadcasting.

### Analysis

Two researchers (SP and KF) scrutinised the data and constructed a coding frame. Interviews were systematically coded using a modified grounded theory approach so that data were explored for well-established as well as emergent themes. Deviant cases were included in the analysis. N6 software was used to facilitate a comparison of themes across the entire dataset. Our analysis reveals new patient perspectives that are unlikely to appear in standard questionnaires or health-related quality of life instruments.

## Results

Participants who had attended at least one ICU follow-up appointment, and those who had not been offered an appointment, discussed their perceptions and experiences during the period after they came home. Analysis identified four main themes: continuity of care; receiving information; importance of expert reassurance; and giving feedback to ICU staff. These will be discussed in turn and illustrated by interview data.

### Continuity of care

Continuity of care after hospital discharge and during recovery was extremely important to participants. Although the form they wanted this care to take varied, it included having tests and being monitored, referrals to other specialists and ICU follow-up appointments soon after hospital discharge and sometimes more than once.

#### Having tests and being monitored

Participants with at least one ICU follow-up appointment had found it helpful because the tests that were conducted in the follow-up reassured them that they were being monitored. The appointment had been a valuable opportunity to discuss their concerns and ensure problems were detected and treated as soon as possible.

#### Being referred

Participants who had been referred to other specialists after their first follow-up appointment had been pleased the problem had been addressed and that they would receive more care. Some had been referred to clinical psychologists, others to physiotherapists or occupational therapists. For participants, part of the continuity of care had also been having the next stage of care organised before hospital discharge and seeing the same health professionals whenever possible: patients valued seeing health professionals they were familiar with, who remembered them and who could see the progress they were making.

I also saw a specialist in behavioural medicine who's part of the follow-up team and she was absolutely brilliant... I don't think I could have done it without her... Each time I went to see her she'd sort of say, you know, "How have you felt this week?" And I'd tell her what had happened and she'd say, "Well next week this will probably happen, you'll probably feel like this." And then when it happened, 'cause she'd prepared me for the anxiety attacks and the panic attacks and then when it happens you don't feel quite that bad because you think, "She's already told me this might happen. And she told me that might happen." So I'm not going daft.

[41-year-old woman, admitted to the ICU because of complications during pregnancy]

#### Timing and frequency of appointments

For those participants who were recovering well, one ICU follow-up appointment was often enough to discuss their concerns and they saw "no point" in having further consultations. They were satisfied with being discharged and consulting their GP if any further problems arose. Many participants, although happy with the opportunity to attend an ICU follow-up appointment, said they would have liked more than one appointment and the first one soon after hospital discharge rather than several months later because this was when they had needed it most. Participants found the first few months after hospital discharge the most difficult. They had needed a lot of reassurance but could feel uncomfortable about phoning ICU nurses for information, even when nurses had encouraged them to do so, because they thought they were too busy and likely to have forgotten them.

I, of course, wasn't at work, still at home recovering... So you spend far too much time chewing the cud and feeling frustrated that you'd like to kind of do something about it. And that's why it was a good thing that they had a follow up. But the follow up came far too long after. It needs to be a lot sooner.

[IC30, a 55-year-old woman admitted to the ICU with epiglottitis and severe sepsis. She had her first ICU follow-up appointment three months after hospital discharge and the second one six months after discharge]

Participants said that, had they had an ICU follow-up appointment one month after hospital discharge, doctors might have seen how weak they still were and referred them for physiotherapy. They often pointed out that, had they not had family to depend on, they might have taken longer to recover, not recovered so well or not recovered at all.

Participants said that, given how ill they had been, how long recovery after critical illness could take and how susceptible they were or felt to further problems, one appointment had not been enough. They felt that regular appointments or an initial phone call after hospital discharge would have been reassuring and an opportunity to discuss any concerns. Several others said they would have benefited from at least one more appointment because, although their physical health had been discussed in the consultation, their emotional and psychological progress had received little attention and they would have valued more support with their psychosocial recovery. Some participants said they would have valued having counselling but, in its absence, had sought out support groups or former ICU patients to discuss their experiences with.

I still feel frustrated and I still find it hard... I still have to take something to make me sleep, because I still find that hard... I find it hard to watch hospital things on television, where there's somebody in intensive care with the machines all around them... I feel so guilty. He [GP] said, "You need to talk to somebody, talk through these feelings." I've only just now said that I will probably need counselling.

[IC24, a 44-year-old woman admitted to the ICU because of pneumonia]

A few participants felt "abandoned" after hospital discharge either because they felt that one ICU follow-up appointment had not been enough or because they had no ICU follow up at all. A minority paid for private physiotherapy and this had a significant impact on their financial and personal lives. Those who had attended one appointment were disappointed that they had received "no feedback" afterwards.

### Receiving information

For participants, ICU follow-up appointments were an invaluable time for discussing their physical, emotional and psychological recovery, what had happened before and during their ICU stay, and for asking questions. This health information and care was seen as important to their overall recovery.

#### Information about physical, emotional and psychological recovery

Specific information about diet, exercise, the length of time it was taking to improve, medication and returning to work were all important. Many participants said that, although they had received general leaflets and information about recovery when they were discharged, they particularly benefited from information specifically about them and their condition. Participants' information needs also included information about their tracheotomy scar, MRSA, mobility, weakness, sex, support and making a complete recovery. They also wanted to know about the probability of becoming critically ill again and worried about this. Those who had been given an ICU diary wished they had been given it before the first follow-up appointment so they could have read it beforehand and been able to ask questions. Many had found it helpful for information and dates, which they could refer to when seeing other doctors including their GP.

It [ICU diary] took me about five minutes to read. And I read that on the bus going to the park-and-ride [laughs]. And I thought, "Why on earth was I not given this, if not before I came for my appointment for the follow-up meeting, just five minutes before I went to the follow-up?" Because it was full of stuff that I had no idea... And if that had been given to me the next seven days before, I could speak to anybody in intensive care and get the answers. I wouldn't have needed that anxiety. If that had just been given to me 10 minutes before that meeting, I could have asked the questions and had the answers. Simple things, silly things like that. But that was so important.

[IC30, a 55-year-old woman admitted to the ICU with epiglottitis and severe sepsis]

#### Information that helped make sense of their experience

For participants, the ICU follow-up appointment had been valuable because the consultant had talked them through their medical notes, which had given them a better understanding of what had happened when they were in the ICU. They found out more about their illness, treatments, tests and progress, including dates. They had valued being able to ask questions about a crucial time in their lives of which they had few, no or only blurred memories. Discussing their dreams and hallucinations and learning more about what had been real or delusion had been important to participants who felt that, without this consultation, they would have been anxious for longer and less able to "move on". Studies have shown that those patients who do not remember anything about their ICU stay experience more PTSD symptoms because they lack facts to explain delusional memories [38]. For some, follow-up appointments were also a convenient time to visit the ICU with a nurse.

The emotional side was very difficult to come to terms with... You can cry uncontrollably and there was no reason for it... You don't know when it's going to start. You don't know when it's going to stop. You don't know how long it's going to go on for. I found this one of the worst things to come to terms with... Somebody asked me did I want to go to ICU unit while I was there and I felt that particular time I was asked, yes, it was a good time for me. It would have been, I was trying to piece things together in my mind. I was trying to put right a jigsaw of my life, if that's the best way to describe it. And I needed to put pieces together to complete, as I was before.

[IC37, a 58-year-old man admitted to the ICU after a road traffic accident]

### Importance of expert reassurance

For participants, ICU follow-up appointments were particularly important for gaining reassurance from experts familiar with their ICU experience. Reassurance came in the form of tests, referrals, and conversations with and specific information from ICU medical staff. Participants said they had felt more reassured when ICU doctors or nurses had told them about the sometimes similar experiences of other patients and when they had prepared them for what was to come. Participants who had problems with sleep, concentration and memory found it invaluable to be reassured that they were progressing in a normal way. When they first came home, many had felt insecure about no longer being in the safe environment of the hospital. The thought of becoming critically ill again or being readmitted to hospital or the ICU frightened many former patients but, when they had been able to discuss their fears and concerns with the ICU follow-up team, they had felt much better able to cope. One woman, who had had two follow-appointments at the time of interview, praised the doctor who had treated her in the ICU and had phoned her two weeks after hospital discharge to ask about her progress.

There are still days even, what are we six, seven months on now, yeah I just couldn't see the point of anything... in my mind I was thinking "Well what's the point of it, we're all going to die anyway?" And I needed to speak to, I went back and spoke to the nurse consultant on ICU and she explained, and I only saw her the once but she explained it's perfectly normal. And that helped, once she said to me, "Loads of people feel like that when they come out of intensive care and you need to be kind and give yourself a bit of time, it will pass."

[IC04, 46-year-old woman admitted to the ICU with surgical complications and septicaemia]

Being able to speak to a health professional who knew the details of their illness and could answer their questions had been extremely important. Several participants said that, when they had visited their GP, the GP had no knowledge of or information about their ICU stay and so could not answer questions about it. Expert reassurance also included having counselling from someone familiar with ICU patients' experiences.

I saw a counsellor privately and then I was also given a couple of sessions through my GP. But I could really have done with a bit more support from professionals who knew. I mean none of those people knew about intensive care. And I think a bit more support from people who have actually worked with people who've been through the intensive care experience would have been really helpful at the time... They'd [counsellors] never really come across anybody like me or if they had maybe one or two other cases so they didn't really know what to look for.

[IC06, a 35-year-old woman admitted to the ICU because of pneumonia, and also developed septicaemia]

### Giving feedback to ICU staff

ICU follow-up appointments also gave patients the chance to give feedback to ICU staff and discuss aspects of care that were of a poor standard. One woman raised her concern about the difference in nursing care on the ICU and ward; another had gained enormous reassurance after discussing her memories of personal care when she had been sedated. One man, whose partner had been disappointed with the hygiene and cleanliness in the ICU and ward, said the follow-up clinic gave his partner the chance to air her views.

It was five weeks I was in intensive care, and a week in HDU [high-dependency unit]. I knew something was wrong when I was sedated but I didn't quite know what it was... and I did think that just, again I'm really pleased it's quite common, I thought I'd been a victim of a sex crime. And apparently what we've talked about, me and the doctors, we think it was because of the personal care you know, touching me in really intimate places. And I'm a very private person that way so they think that was the link and that made sense.

[IC04, 46-year-old woman admitted to the ICU with surgical complications and septicaemia]

Participants who had been invited to attend an ICU follow-up appointment sometimes felt their feedback would help ICU staff with research into service provision and audit, and many were keen to "give something back". ICU follow up was also an opportunity to see staff again and thank them for the care and support they had provided at an extremely traumatic time.

#### No ICU follow-up

Some participants said they had received no ICU follow-up care but had been followed up in another hospital department, particularly after surgery. Participants felt that the lack of ICU follow up meant that they had no opportunity to be monitored or referred quickly if they had any problems, to find out the details of their illness and ICU stay, or to ask questions. Others, who had no surgery, were upset when they had no follow up at all after being discharged from hospital, despite having been critically ill only months earlier.

We're not sure if the drugs or the respiratory problem has caused the problems with me eyes. So we've got an issue there really. But if you're that bad and then you're let out, are you fit? As I've said, I've got an infection on my chest. Now I worry about those now, for obvious reasons. ... The follow up is pretty abysmal quite honestly. I understand the constraints... An aftercare telephone call, an initial aftercare telephone call only takes one of those people just to say, "I'm just ringing on behalf of so-and-so, just checking to make sure that everything's okay. If it's not, you know, if you've got concerns..." I mean she can read it off a piece of paper. "If you've got concerns, then, you know, I may have to pass you up to somebody else."

[IC26, a 47-year-old man admitted to the ICU with pneumonia]

Not everyone chose to attend ICU follow-up appointments when invited. One woman said she had an extremely difficult time in the ICU, had felt very paranoid and had not wanted to visit the ICU again, but did visit her GP. A few said that they had seen their GP, who had carried out any necessary tests or said that they were recovering well. Others were reluctant to travel to an ICU a long way from their home.

## Discussion

ICU follow-up services have been advocated in UK policy recommendations, although the provision of these services has been inconsistent. Additionally, the clinical benefits and cost-effectiveness of ICU follow-up services are unclear. This study explores patients' perceptions and experiences of these services. The views of patients are important because they shed light on the value of specific services from the perspective of service users themselves. This is the first large qualitative study to focus solely on patients' experiences of ICU follow-up services across the UK. Apart from the depth of our qualitative interview data, the main strengths of this study lie in our large sample size of patients from across the UK, with different age groups, social and ethnic backgrounds and reasons for ICU admission. The qualitative researcher was also unconnected to the ICU service, making it easier for people to speak freely about their experiences.

The limitations of our study are that participants had received ICU follow-up care several months (in some cases years) before the interview and so there may be some recall bias. However, the accounts are very detailed and many of the reported experiences feature in several accounts, reinforcing the findings. The study is not longitudinal, and consisted of 40 interviews designed to capture detailed illness narratives. Because interviews took place at different stages of people's recovery, however, we have been able to show through our analysis how patients' views and needs can and do change over time.

This study highlights that many former patients value having ICU follow-up services but that their healthcare needs are often unmet because many hospitals do not provide this aftercare. When there is no ICU follow-up care, patients can feel that health professionals are no longer interested in them, can wonder why they are taking so long to recover or whether they were really so ill in the first place. This can lead to unrealistic expectations and patients trying to do too much too soon, as well as frustration, anxiety or depression because of the pace of recovery. No memory of the ICU stay has also been associated with more PTSD symptoms. Information given at the ICU follow-up appointment tells patients of the gravity of their illness, of how far they have come since hospital admission, and of realistic goal setting for and expectations of their recovery. Assessing whether an out-patient needs further tests or a referral at an ICU follow-up appointment can also lead to improved health outcomes and cost-saving in the long run. ICU follow up and feedback from patients can provide data on mortality and health-related quality of life measures after ICU stay and hospital discharge, and data for monitoring service provision. Many participants valued giving feedback to ICU staff about their experiences of healthcare and quality of life after ICU treatment, data not only important for audit and research but also to the overall job satisfaction and morale of ICU nurses [39].

Although patients value ICU follow-up services, it is still unclear whether they change patient outcomes. Research currently underway aims to evaluate quantitatively whether ICU follow-up services are cost-effective in terms of outcome [22]. Most participants were aware of the financial constraints on the health system and, although they valued ICU follow-up care, they did not want it to continue indefinitely, many of them declining appointment invitations when they themselves felt they no longer needed them.

Although this study is not a service evaluation, ICU clinicians could reconsider their practice particularly in terms of continuity of care and information provision. An initial phone call shortly after hospital discharge was viewed by participants as extremely important, not only in terms of gaining reassurance but also to be able to ask basic questions that were causing, often unnecessary, anxiety. A phone call (or email) shortly after hospital discharge by ICU clinicians could be used to check how each patient is managing and help identify possible problems at an early stage. Specific information about recovery was particularly important to former ICU patients as was, often, the experiences of others. Patients and carers could be routinely told verbally and in writing how to contact local support groups and website resources such as the Healthtalkonline website [30] to enable them to look for support and information for themselves.

## Conclusions

Although many former ICU patients received informal support from various sources, including family and friends, ICU follow-up services were seen by participants as an important contribution to their physical, emotional and psychological recovery in terms of continuity of care, receiving information, gaining expert reassurance and giving feedback to ICU staff. For participants, this service is best provided and often could only be provided by those who were familiar with the details of their ICU stay, and able to answer questions and offer clarification.

Further research is also needed on the follow-up needs of specific groups of ICU patients, including long/short stay patients and those admitted for specific conditions such as brain injury. ICU clinicians could also look to cancer research for guidance on follow up, where a successful multidisciplinary approach has been used for over 50 years.

This study demonstrates that data generated by qualitative interviews could be important to ICU clinicians interested in learning more about the perceptions and experiences of ICU patients. For participants, the service users, ICU follow-up care was an important service in terms of continuity of care after critical illness and, in turn, their overall physical, emotional and psychological recovery.

## Key messages

• Patients value having ICU follow-up services but their healthcare needs are often not met because many hospitals are unable to provide the aftercare they require.

• Patients view ICU follow-up services as an important contribution to their physical, emotional and psychological recovery in terms of continuity of care, receiving information, gaining expert reassurance and giving feedback to ICU health professionals about the care they received.

• Continuity of care is important to patients in terms of having tests and being monitored, receiving referrals and ICU follow-up appointments soon after hospital discharge.

• Patients without access to ICU follow-up services often feel abandoned or disappointed because they have no opportunity to get feedback on their progress, be referred quickly if they are having problems or find out the details of their illness and ICU stay.

• Most patients are aware of the financial constraints on the health system and, although they value ICU follow-up care, they do not want it to continue indefinitely, with many of them declining appointment invitations when they themselves feel they no longer need them.

## Abbreviations

DIPEx: Database of Individual Patient Experiences; ICNARC: Intensive Care National Audit and Research Centre; ICU: intensive care unit; NHS: National Health Service; PTSD: post-traumatic stress disorder.

## Competing interests

The authors declare that they have no competing interests. This work was supported by the Intensive Care National Audit and Research Centre (ICNARC). The authors' work was conducted independently of the funding body.

## Authors' contributions

SP interviewed the patients and analysed the data together with KF. SP drafted the paper; all authors contributed to subsequent drafts and the final version.
